# From Death Triad to Death Tetrad—The Addition of a Hypotension Component to the Death Triad Improves Mortality Risk Stratification in Trauma Patients: A Retrospective Cohort Study

**DOI:** 10.3390/diagnostics12112885

**Published:** 2022-11-21

**Authors:** Wei-Juo Tzeng, Hsiang-Yu Tseng, Teng-Yuan Hou, Sheng-En Chou, Wei-Ti Su, Shiun-Yuan Hsu, Ching-Hua Hsieh

**Affiliations:** 1 Department of General Surgery, Kaohsiung Chang Gung Memorial Hospital and Chang Gung University College of Medicine, Kaohsiung 83301, Taiwan; 2 Department of Trauma Surgery, Kaohsiung Chang Gung Memorial Hospital and Chang Gung University College of Medicine, Kaohsiung 83301, Taiwan

**Keywords:** coagulopathy, hypothermia, acidosis, hypotension, death triad, death tetrad, mortality, trauma

## Abstract

The death triad, including coagulopathy, hypothermia, and acidosis, is shown to be a strong predictor of mortality in trauma patients. We aimed to investigate whether the inclusion of hypotension, defined as systolic blood pressure (SBP) < 60 mmHg, as a fourth factor in the death triad would comprise a death tetrad to help stratify mortality risk in trauma patients. A total of 3361 adult trauma patients between 1 January 2009 and 31 December 2019 were allocated into groups to investigate whether hypotension matters in determining the mortality outcome of trauma patients who possess 1–3 death triad components compared to those without any component. Hypotension was added to the death tetrad, and the adjusted mortality outcome was compared among groups with 0–4 death tetrad components. Herein, we showed that SBP < 60 mmHg could be used to identify patients at risk of mortality among patients with one or two death triad components. Patients with one, two, and three death tetrad components had respective adjusted mortality rates of 3.69-, 10.10-, and 40.18-fold, determined by sex, age, and comorbidities. The mortality rate of trauma patients with all the four death tetrad components was 100%. The study suggested that hypotension, defined as an SBP < 60 mmHg, may act as a proper death tetrad component to stratify the mortality risk of trauma patients.

## 1. Introduction

The death triad includes metabolic acidosis in full blood (potential of hydrogen (PH) < 7.2), hypothermia (temperature < 35 °C measured by tympanic thermometers), and coagulopathy (international normalized ratio [INR] > 1.5) when patients arrive at the emergency department. The death triad observed in trauma patients has been proven to be a strong predictor of mortality [1,2,3,4]. The mortality of trauma patients at 24 h increased with the addition of all three components of the death triad [5]. In patients with multiple traumas, the death triad predicted 24 h mortality in 96% of patients [6]. In patients with abdominal gunshot wounds and major vascular injuries whose mortality rate was as high as 40%, the death triad comprised 85% of mortality [7]. Mitra et al. found that the overall mortality rate would be as high as 47.8% if the trauma patient presented with all three components of the death triad [1]. Thus, the death triad has been used worldwide and it provides important information for risk stratification in dealing with trauma patients.

Each component of the death triad had a negative impact on patient survival, and the relationship between the death triad and mortality was clear and validated [1,8]. Among the three components of the death triad, metabolic acidosis has been proven to be associated with increased mortality [9], coagulopathy was related to a 4- to 5-fold increase in mortality following major trauma [10], and hypothermia contributes not only to mortality associated with the injury but also to fluid requirements and duration of surgery [7,11]. Furthermore, hypothermia is also a significant contributor to coagulopathy, regardless of the condition of metabolic acidosis or fluid infusion [12,13].

Interestingly, although hypotension has been widely recognized as a major contributor of mortality in trauma patients [14,15,16,17,18], the death triad did not use hypotension as a component in determining patient outcomes. This study aimed to investigate whether hypotension matters in determining the mortality outcome of trauma patients with various components of the death triad and to explore whether hypotension could act as a fourth factor besides the death triad to stratify the mortality risk in trauma patients.

## 2. Materials and Methods

### 2.1. Study Population and Grouping

**Figure 1:**
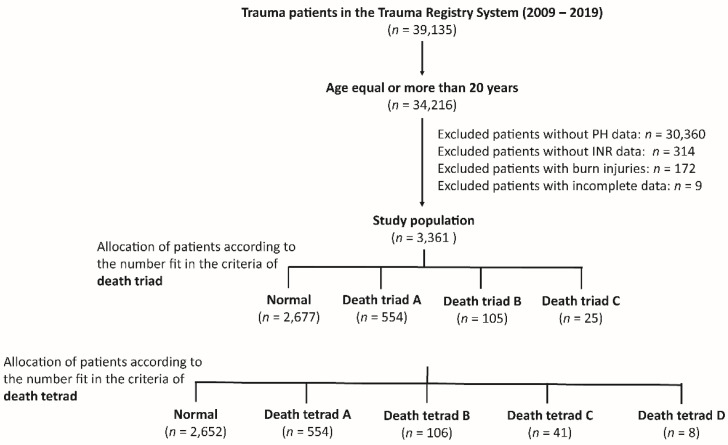
Flowchart illustrating the included hospitalized adult trauma patients from the registered trauma database, and the assignment of the study patient populations into three or four groups according to the number fit in the components of death triad or tetrad, respectively. In comparison with the death triad, the death tetrad has an additional component, hypotension, defined as systolic blood pressure < 60 mmHg. PH, potential of hydrogen; INR, international normalized ratio.

This study reviewed registered medical information from the registered trauma database between 1 January 2009 and 31 December 2019 in a Level I trauma center that provides care to trauma patients in southern Taiwan [19,20,21]. All registered data were prospectively collected from two licensed registers of hospitalized trauma patients. As shown in Figure 1, the eligibility of 39,195 hospitalized trauma patients was assessed. After excluding those aged <20 years, those lacking PH data (*n* = 30,360), lack of INR data (*n* = 314), patients with burn injuries (*n* = 172), and those with incomplete registered data (*n* = 9), 3361 patients were enrolled in the study for further analysis. First, trauma patients were grouped according to number fit in the components of the death triad. Patients with one, two, and three items fit in the components of the death triad and were allocated into death triad groups A, B, and C, respectively (Figure 1). With hypotension, defined as a non-invasive measurement of systolic blood pressure (SBP) < 60 mmHg at the triage station when patients arrived at the emergency department, as one component of the death tetrad, further grouping was performed according to the number fit in the components of the death tetrad. Patients with one, two, three, and four items fit in the components of the death tetrad and were allocated into the death tetrad groups A, B, C, and D, respectively (Figure 1).

### 2.2. Study Parameter

The study included information on age, sex, comorbidities (including coronary artery disease (CVA), congestive heart failure (CHF), hypertension (HTN), diabetes mellitus (DM), and end-stage renal disease (ESRD)), vital signs, PH, INR, Glasgow Coma Scale (GCS) score, abbreviated injury scale (AIS) in each body region, injury severity score (ISS), in-hospital mortality, length of hospital stay (LOS), and admission into the intensive care unit (ICU). The polytrauma is defined as the trauma patients have injuries of AIS ≥ 3 in more than one body region [22,23].

### 2.3. Statistical Analysis

Data processing and analysis were performed using IBM SPSS Statistics for Windows (version 20.0; IBM Corp., Armonk, NY, USA). Pearson chi-square tests were employed to compare categorical variables and are presented as frequencies and percentages with odds ratios (OR) and 95% confidence intervals (CI). Continuous variables were estimated using Levene’s test for homogeneity of variance. Normally distributed continuous variables are presented as mean and standard deviation, and the unpaired Student’s *t*-test was used to compare continuous variables that were normally distributed. The Mann–Whitney *U* test was used to compare non-normally distributed data, which are presented as medians and interquartile ranges (IQR). In this study, in-hospital mortality was the primary outcome, while hospital LOS and ICU admission were the secondary outcomes. The adjusted odds ratio of mortality was computed using logistic regression adjusted for variables with significant differences in patients’ injury characteristics. Statistical significance was set at a calculated *p*-value of <0.05.

## 3. Results

### 3.1. Clinical Characteristics and Outcomes of Trauma Accident Divided According to Death Triad

According to the death triad, the trauma patients were divided into four groups: death triad A group (*n* = 554), which are those who fulfilled one component of the death triad; death triad B (*n* = 105) and death triad C (*n* = 25) groups, which are those who fulfilled two and three components of the death triad, respectively. The remaining patients were allocated to a normal group (*n* = 2677). As shown in Table 1, the groups of patients showed significant differences in sex, age, and comorbidities of CVA, HTN, and DM. Regarding the consciousness level, the group with more components of the death triad had a lower GCS score than those in the normal group. The median (IQR) GCS of death triad A group was 9 (4–15), death triad B was 4 (3–8), and death triad C was 3 (3–7), in comparison with those in the normal group, which was 15 (8–15). Compared with those patients in the normal group, the group who had component(s) of the death triad had a higher percent of patients with AIS ≥ 3 injuries in the head, thorax, and abdomen as well as patients with polytrauma. Additionally, the group who had more components of the death triad tended to have a higher ISS compared with those in the normal group. The median (IQR) ISS of death triad A group was 22 (16–29), death triad B was 25 (21–34), and death triad C was 25 (17–38), in comparison with those in the normal group, which was 16 (924). With the increased mortality rate for patients with more components of the death triad, the mortality rate in the death triad A (29.1%), B (57.1%), and C (84.0%) groups was significantly higher than that in the normal group (10.6%, all *p* < 0.001). The requirement for admission into the ICU was higher for those with components of the death triad than for those in the normal group (*p* < 0.001), while in the hospital LOS, there was no significant difference among these groups (*p* = 0.060).

### 3.2. Outcomes of Trauma Patients with or without Hypotension and Those with One to Three Components of the Death Triad

This study investigated whether hypotension is important in determining the mortality outcome of trauma patients with one to three components of the death triad. Patients in the death triad A, B, and C groups were divided into those with SBP < 60 mmHg and those with SBP ≥ 60 mmHg. Among the death triad A group patients (Table 2), there was no difference in sex, age, comorbidities, and ISS between the patients with SBP < 60 mmHg and those with SBP ≥ 60 mmHg; however, the patients with SBP < 60 mmHg had a lower GCS (median, IQR: 3 (3–6) vs. 9 (4–15), *p* < 0.001) and higher mortality rate than those with SBP ≥ 60 mmHg (48.0% vs. 28.2%, *p* = 0.033). No differences in hospital LOS and ICU admission rates were found between the two groups of patients.

Among the death triad B patients (Table 3), there was no difference in age, comorbidities, GCS, and ISS between the patients with SBP < 60 mmHg and those with SBP ≥ 60 mmHg; however, the patients with SBP < 60 mmHg predominantly had more males and higher mortality rates than those with SBP ≥ 60 mmHg (79.2% vs. 50.6%, *p* = 0.013). No differences in hospital LOS and ICU admission rates were found between the two groups of patients.

Among the death triad C patients (Table 4), there was no difference in sex, age, comorbidities, GCS, and ISS between the patients with SBP < 60 mmHg and those with SBP ≥ 60 mmHg. The mortality rate (100.0% vs. 76.5%, *p* = 0.134), hospital LOS, and ICU admission rate were not significantly different between the death triad C patients with SBP < 60 mmHg and those with SBP ≥ 60 mmHg.

### 3.3. Clinical Characteristics and Outcomes of Trauma Accidents with Different Death Tetrad Score

Based on our hypothesis, we added SBP < 60 mmHg as the fourth component to stratify the risk of mortality. According to the death tetrad, the trauma patients can be divided into five groups: death tetrad A (*n* = 554) group, those who fulfilled one component of the death tetrad; death tetrad B (*n* = 106), C (*n* = 41), and D (*n* = 8) groups, those who fulfilled two, three, and four components of the death tetrad, respectively. The remaining patients were allocated to the normal group (*n* = 2652). These groups of patients were significantly different in terms of sex, age, and comorbidities of CVA, HTN, CHF, DM, GCS, and ISS (Table 5). With the increased mortality rate for those patients with more components of the death tetrad, the mortality rate in the death tetrad A (28.2%), B (50.0%), C (78.0%), and D (100%) groups was significantly higher than that in the normal group (10.4%, all *p* < 0.001). Controlling for the underlying patient characteristics of sex, age, and comorbidities (CVA, HTN, CHF, and DM), patients in the death tetrad A, B, and C groups had a respective adjusted mortality rate of 3.69-, 10.10-, and 40.18-fold than those in the normal group. There was 100% mortality for all the eight trauma patients with all four components of the death tetrad. The requirement for admission into the ICU was higher for those with components of the death tetrad than for those in the normal group (*p* < 0.001), while there was no significant difference in the hospital LOS among these groups of patients (*p* = 0.052).

## 4. Discussion

This study revealed that SBP < 60 mmHg could be further used to identify patients at risk of mortality with one or two components of the death triad. However, there was no significant difference in the mortality rate for those who had all components of the death triagles. This may be due to the relatively small number of patients. The study also suggested that an SBP < 60 mmHg may act as a proper component of the death tetrad of trauma patients to stratify the risk of mortality. The mortality rate of trauma patients with all three components of the death triad was 84%, while in patients with all four components of the death tetrad, the mortality rate was 100%. In addition to coagulopathy, hypothermia, and acidosis, the addition of hypotension as a death tetrad may be applied for mortality risk stratification in patients with trauma.

To improve the prediction of mortality in trauma patients, the addition of hypocalcemia on the lethal triage had been proposed to become “lethal diamond” [24]. The study found that a lower calcium level on admission to the emergency department was associated with increased mortality [25]. Calcium is also thought to play a key role in resuscitation [24]. However, the measurement of calcium level is not routinely performed, and it takes time to obtain the result of serum calcium levels. Additionally, although ISS was able to predict mortality [26] and the necessity for hospitalization and ICU [27], ISS is not suitable as a predictor for patients’ outcome assessment in the emergency room because ISS was calculated only after a whole-body examination was performed and was not immediately available in the emergency room. However, GCS may be considered as a choice for add-on in the death triad to stratify patients who are at risk of mortality. One study used GCS instead of temperature as the BIG (base deficit [B], international normalized ratio [I], and GCS [G]) score for predicting mortality in pediatric trauma [23]. The predictive power was comparable with that of more complex systems, such as pediatric logistic organ dysfunction, Pediatric Index of Mortality 2, and Pediatric Risk of Mortality III [28]. However, most GCS evaluations may be reserved for patients with head injury. In contrast, SBP could be detected in patients injured by all causes of trauma and is easily monitored repeatedly, non-invasively, inexpensively, and rapidly.

This study has some limitations. First, there was a small number of trauma patients who had all components of the death tetrad. Second, selection bias may exist due to the retrospective nature of the study. Additionally, because this study only evaluated in-hospital mortality but not long-term mortality, a selection bias may have occurred in the outcome measurement. Moreover, some pre-hospital interventions, such as damage control, blood and fluid transfusion, and resuscitation, may result in different patient outcomes, and these factors were not controlled for further analysis. Additionally, the definition of hypotension with SBP < 60 mmHg is arbitrary, and the regular blood pressure of the patients prior to the trauma injury is unknown. The study regarding how many of those hypotensive patients who develop the worst outcome have an increase in circulating lactate and an alteration in the base excess may help illustrate the relationship of these components of tetrad [29]; however, the lack of lactate data in most of the patient population deterred such an investigation in this study. Furthermore, the inclusion of the patients with different proportions of injuries to each body region may lead to bias in the outcome measurement. Finally, the results of this study were limited to a single urban trauma center, which may not be generalizable to other areas.

## 5. Conclusions

The study suggested that hypotension, defined as an SBP < 60 mmHg, may act as a proper component of the death tetrad to stratify the mortality risk of trauma patients.

## Figures and Tables

**Table 1:** The trauma patients grouped according to the number fit in the components of the death triad. The patients with one, two, and three items that fit in the components of the death triad were allocated into the group of death triad A, B, C, respectively.

Variables	Grouping by Number of Components of Death Triad				
	Death Triad A *n* = 554	Death Triad B *n* = 105	Death Triad C *n* = 25	Normal *n* = 2677	*p*
Gender					<0.001
Male, *n* (%)	385 (69.5)	90 (85.7)	20 (80.0)	1698 (63.4)	
Female, *n* (%)	169 (30.5)	15 (14.3)	5 (20.0)	979 (36.6)	
Age, years (SD)	53.8 ± 20.3	52.7 ± 18.8	47.2 ± 18.4	57.6 ± 19.9	<0.001
Comorbidities					
CVA, *n* (%)	14 (2.5)	1 (1.0)	0 (0.0)	156 (5.8)	0.001
HTN, *n* (%)	159 (28.7)	24 (22.9)	5 (20.0)	967 (36.1)	<0.001
CAD, *n* (%)	41 (7.4)	4 (3.8)	0 (0.0)	173 (6.5)	0.291
CHF, *n* (%)	8 (1.4)	0 (0.0)	1 (4.0)	21 (0.8)	0.115
DM, *n* (%)	84 (15.2)	10 (9.5)	1 (4.0)	543 (20.3)	<0.001
ESRD, *n* (%)	18 (3.2)	1 (1.0)	1 (4.0)	108 (4.0)	0.364
GCS, median (IQR)	9 (4–15)	4 (3–8)	3 (3–7)	15 (8–15)	<0.001
Head AIS ≥ 3, *n* (%)	345 (62.3)	70 (66.7)	16 (64.0)	1497 (55.9)	0.008
Thorax AIS ≥ 3, *n* (%)	153 (27.6)	43 (41.0)	12 (48.0)	519 (19.4)	<0.001
Abdomen AIS ≥ 3, *n* (%)	75 (13.5)	22 (21.0)	8 (32.0)	154 (5.8)	<0.001
Extremities AIS ≥ 3, *n* (%)	132 (23.8)	28 (26.7)	4 (16.0)	652 (24.4)	0.723
External AIS ≥ 3, *n* (%)	0 (0.0)	0 (0.0)	0 (0.0)	1 (0.0)	0.968
Polytrauma, *n* (%)	161 (29.1)	51 (48.6)	13 (52.0)	418 (15.6)	<0.001
ISS, median (IQR)	22 (16–29)	25 (21–34)	25 (17–38)	16 (9–24)	<0.001
1–15, *n* (%)	131 (23.6)	9 (8.6)	5 (20.0)	1066 (39.8)	<0.001
16–24, *n* (%)	165 (29.8)	28 (26.7)	4 (16.0)	956 (35.7)	0.003
≥25, *n* (%)	258 (46.6)	68 (64.8)	16 (64.0)	655 (24.5)	<0.001
Mortality, *n* (%)	161 (29.1)	60 (57.1)	21 (84.0)	283 (10.6)	<0.001
Hospital LOS, days (SD)	18.6 ± 16.9	17.7 ± 20.2	13.5 ± 18.8	16.7 ± 15.7	0.060
Admitted into ICU, *n* (%)	469 (84.7)	99 (94.3)	19 (76.0)	1793 (67.0)	<0.001

CAD = coronary artery disease; CHF = congestive heart failure; CI = confidence interval; CVA = cerebral vascular accident; DM = diabetes mellitus; ESRD = end-stage renal disease; GCS = Glasgow Coma Scale; HTN = hypertension; ICU = intensive care unit; IQR = interquartile range; ISS = injury severity score; LOS = length of stay; OR= odds ratio; SD = standard deviation.

**Table 2:** The injury characteristics and outcome of trauma patients in death triad A with or without systolic blood pressure less than 60 mmHg.

Variables	Death Triad A			
	SBP < 60 mmHg *n* = 25	SBP ≥ 60 mmHg *n* = 529	OR (95% CI)	*p*
Gender				0.243
Male, *n* (%)	20 (80.0)	365 (69.0)	1.80 (0.66–4.87)	
Female, *n* (%)	5 (20.0)	164 (31.0)	0.56 (0.21–1.51)	
Age, years (SD)	52.7 ± 17.8	53.8 ± 20.4	-	0.787
Comorbidities				
CVA, *n* (%)	1 (4.0)	13 (2.5)	1.65 (0.21–13.17)	0.631
HTN, *n* (%)	7 (28.0)	152 (28.7)	0.97 (0.40–2.36)	0.937
CAD, *n* (%)	1 (4.0)	40 (7.6)	0.51 (0.07–3.86)	0.506
CHF, *n* (%)	0 (0.0)	8 (1.5)	-	0.536
DM, *n* (%)	3 (12.0)	81 (15.3)	0.75 (0.22–2.58)	0.652
ESRD, *n* (%)	1 (4.0)	17 (3.2)	1.26 (0.16–9.83)	0.828
GCS, median (IQR)	3 (3–6)	9 (4–15)	-	<0.001
ISS, median (IQR)	25 (17–28)	22 (16–29)	-	0.245
1–15, *n* (%)	4 (16.0)	127 (24.0)	0.60 (0.20–1.79)	0.357
16–24, *n* (%)	7 (28.0)	158 (29.9)	0.91 (0.37–2.23)	0.842
≥25, *n* (%)	14 (56.0)	244 (46.1)	1.49 (0.66–3.34)	0.333
Mortality, *n* (%)	12 (48.0)	149 (28.2)	2.35 (1.05–5.28)	0.033
Hospital LOS, days (SD)	18.0 ± 18.0	18.6 ± 16.8	-	0.873
Admitted into ICU, *n* (%)	20 (80.0)	449 (84.9)	0.71 (0.26–1.95)	0.508

CAD = coronary artery disease; CHF = congestive heart failure; CI = confidence interval; CVA = cerebral vascular accident; DM = diabetes mellitus; ESRD = end-stage renal disease; GCS = Glasgow Coma Scale; HTN = hypertension; IQR = interquartile range; ISS = injury severity score; OR= odds ratio; SBP = systolic blood pressure; SD = standard deviation.

**Table 3:** The injury characteristics and outcome of trauma patients in death triad B with or without systolic blood pressure less than 60 mmHg.

Variables	Death Triad B			
	SBP < 60 mmHg *n* = 24	SBP ≥ 60 mmHg *n* = 81	OR (95% CI)	*p*
Gender				0.018
Male, *n* (%)	17 (70.8)	73 (90.1)	0.27 (0.09–0.84)	
Female, *n* (%)	7 (29.2)	8 (9.9)	3.76 (1.20–11.79)	
Age, years (SD)	54.8 ± 17.6	52.1 ± 19.2	-	0.541
Comorbidities				
CVA, *n* (%)	0 (0.0)	1 (1.2)	-	0.584
HTN, *n* (%)	8 (33.3)	16 (19.8)	2.03 (0.74–5.58)	0.164
CAD, *n* (%)	0 (0.0)	4 (4.9)	-	0.267
CHF, *n* (%)	0 (0.0)	0 (0.0)	-	-
DM, *n* (%)	3 (12.5)	7 (8.6)	1.51 (0.36–6.35)	0.572
ESRD, *n* (%)	0 (0.0)	1 (1.2)	-	0.584
GCS, median (IQR)	3 (3–6)	5 (3–9)	-	0.230
ISS, median (IQR)	25 (17–36)	25 (22–34)	-	0.296
1–15, *n* (%)	4 (16.7)	5 (6.2)	3.04 (0.75–12.38)	0.107
16–24, *n* (%)	6 (25.0)	22 (27.2)	0.89 (0.31–2.54)	0.833
≥25, *n* (%)	14 (58.3)	54 (66.7)	0.70 (0.28–1.78)	0.453
Mortality, *n* (%)	19 (79.2)	41 (50.6)	3.71 (1.26–10.89)	0.013
Hospital LOS, days (SD)	14.8 ± 21.2	18.5 ± 20.0	-	0.426
Admitted into ICU, *n* (%)	24 (100)	75 (92.6)	-	0.170

CAD = coronary artery disease; CHF = congestive heart failure; CI = confidence interval; CVA = cerebral vascular accident; DM = diabetes mellitus; ESRD = end-stage renal disease; GCS = Glasgow Coma Scale; HTN = hypertension; IQR = interquartile range; ISS = injury severity score; OR= odds ratio; SBP = systolic blood pressure; SD = standard deviation.

**Table 4:** The injury characteristics and outcome of trauma patients in death triad C with or without systolic blood pressure less than 60 mmHg.

Variables	Death Triad C			
	SBP < 60 mmHg *n* = 8	SBP ≥ 60 mmHg *n* = 17	OR (95% CI)	*p*
Gender				0.668
Male, *n* (%)	6 (75.0)	14 (82.4)	0.64 (0.09–4.89)	
Female, *n* (%)	2 (25.0)	3 (17.6)	1.56 (0.21–11.83)	
Age, years (SD)	52.3 ± 16.4	44.8 ± 19.4	-	0.359
Comorbidities				
CVA, *n* (%)	0 (0.0)	0 (0.0)	-	-
HTN, *n* (%)	2 (25.0)	3 (17.6)	1.56 (0.21–11.83)	0.668
CAD, *n* (%)	0 (0.0)	0 (0.0)	-	-
CHF, *n* (%)	1 (12.5)	0 (0.0)	-	0.137
DM, *n* (%)	0 (0.0)	1 (5.9)	-	0.484
ESRD, *n* (%)	1 (12.5)	0 (0.0)	-	0.137
GCS, median (IQR)	3 (3–3)	3 (3–12)	-	0.048
ISS, median (IQR)	25 (18–37)	29 (15–38)	-	0.725
1–15, *n* (%)	1 (12.5)	4 (23.5)	0.46 (0.04–5.00)	0.520
16–24, *n* (%)	2 (25.0)	2 (11.8)	2.50 (0.28–22.04)	0.400
≥25, *n* (%)	5 (62.5)	11 (64.7)	0.91 (0.16–5.20)	0.915
Mortality, *n* (%)	8 (100)	13 (76.5)	-	0.134
Hospital LOS, days (SD)	7.1 ± 10.1	16.5 ± 21.3	-	0.151
Admitted into ICU, *n* (%)	4 (50.0)	15 (88.2)	0.13 (0.02–1.01)	0.037

CAD = coronary artery disease; CHF = congestive heart failure; CI = confidence interval; CVA = cerebral vascular accident; DM = diabetes mellitus; ESRD = end-stage renal disease; GCS = Glasgow Coma Scale; HTN = hypertension; IQR = interquartile range; ISS = injury severity score; OR= odds ratio; SBP = systolic blood pressure; SD = standard deviation.

**Table 5:** The trauma patients grouped according to the number fit in the components of death tetrad. The patients with one, two, three, four items that fit in the components of the death tetrad were allocated into the group of death tetrad A, B, C, D, respectively.

Variables	Trauma Death Tetrad					
	Death Tetrad A *n* = 554	Death Tetrad B *n* = 106	Death Tetrad C *n* = 41	Death Tetrad D *n* = 8	Normal *n* = 2652	*p*
Gender						<0.001
Male, *n* (%)	381 (68.8)	93 (87.7)	31 (75.6)	6 (75.0)	1682 (63.4)	
Female, *n* (%)	173 (31.2)	13 (12.3)	10 (24.4)	2 (25.0)	970 (36.6)	
Age, years (SD)	53.7 ± 20.3	52.2 ± 18.8	50.6 ± 18.8	52.3 ± 16.4	57.6 ± 19.9	<0.001
Comorbidities						
CVA, *n* (%)	13 (2.3)	2 (1.9)	0 (0.0)	0 (0.0)	156 (5.9)	0.002
HTN, *n* (%)	157 (28.3)	23 (21.7)	11 (26.8)	2 (25.0)	962 (36.3)	<0.001
CAD, *n* (%)	42 (7.6)	5 (4.7)	0 (0.0)	0 (0.0)	171 (6.4)	0.282
CHF, *n* (%)	8 (1.4)	0 (0.0)	0 (0.0)	1 (12.5)	21 (0.8)	0.003
DM, *n* (%)	83 (15.0)	10 (9.4)	4 (9.8)	0 (0.0)	541 (20.4)	0.001
ESRD, *n* (%)	18 (3.2)	2 (1.9)	0 (0.0)	1 (12.5)	107 (4.0)	0.269
GCS, median (IQR)	9 (4–15)	4 (3–8)	3 (3–9)	3 (3–3)	15 (8–15)	<0.001
ISS, median (IQR)	22 (16–29)	25 (21–33)	25 (16–38)	25 (18–37)	16 (9–24)	<0.001
1–15, *n* (%)	134 (24.2)	9 (8.5)	8 (19.5)	1 (12.5)	1059 (39.9)	<0.001
16–24, *n* (%)	164 (29.6)	29 (27.4)	8 (19.5)	2 (25.0)	950 (35.8)	0.005
≥25, *n* (%)	256 (46.2)	68 (64.2)	25 (61.0)	5 (62.5)	643 (24.2)	<0.001
Mortality(%)	156 (28.2)	53 (50.0)	32 (78.0)	8 (100)	276 (10.4)	<0.001
AOR of Mortality	3.69 (2.93–4.65)	10.10 (6.65–15.35)	40.18 (18.73–86.22)	-	-	<0.001
Hospital LOS, days (SD)	18.4 ± 16.8	18.4 ± 19.5	15.5 ± 21.0	7.1 ± 10.1	16.7 ± 15.6	0.052
Admitted into ICU, *n* (%)	468 (84.5)	95 (89.6)	39 (95.1)	4 (50.0)	1774 (66.9)	<0.001

AOR= Adjusted odds ratio; CAD = coronary artery disease; CHF = congestive heart failure; CI = confidence interval; CVA = cerebral vascular accident; DM = diabetes mellitus; ESRD = end-stage renal disease; GCS = Glasgow Coma Scale; HTN = hypertension; IQR = interquartile range; ISS = injury severity score; OR= odds ratio; SD = standard deviation.

## Data Availability

Not applicable.
